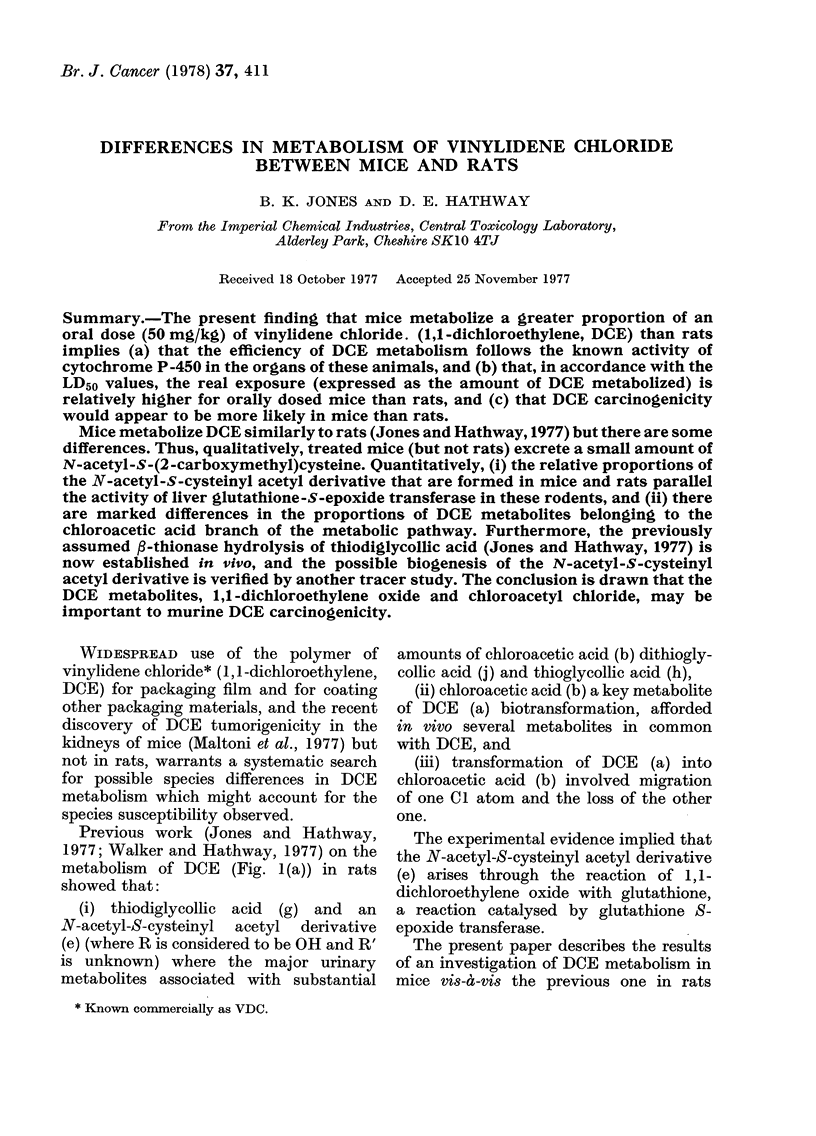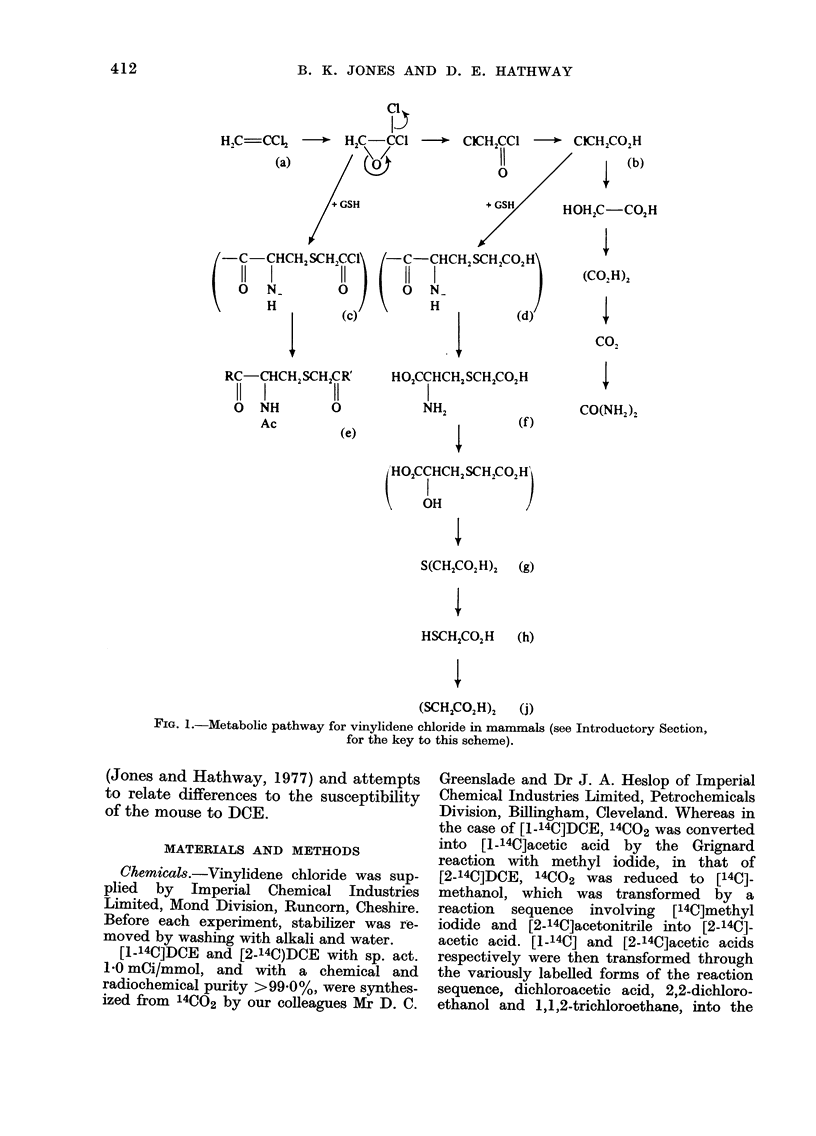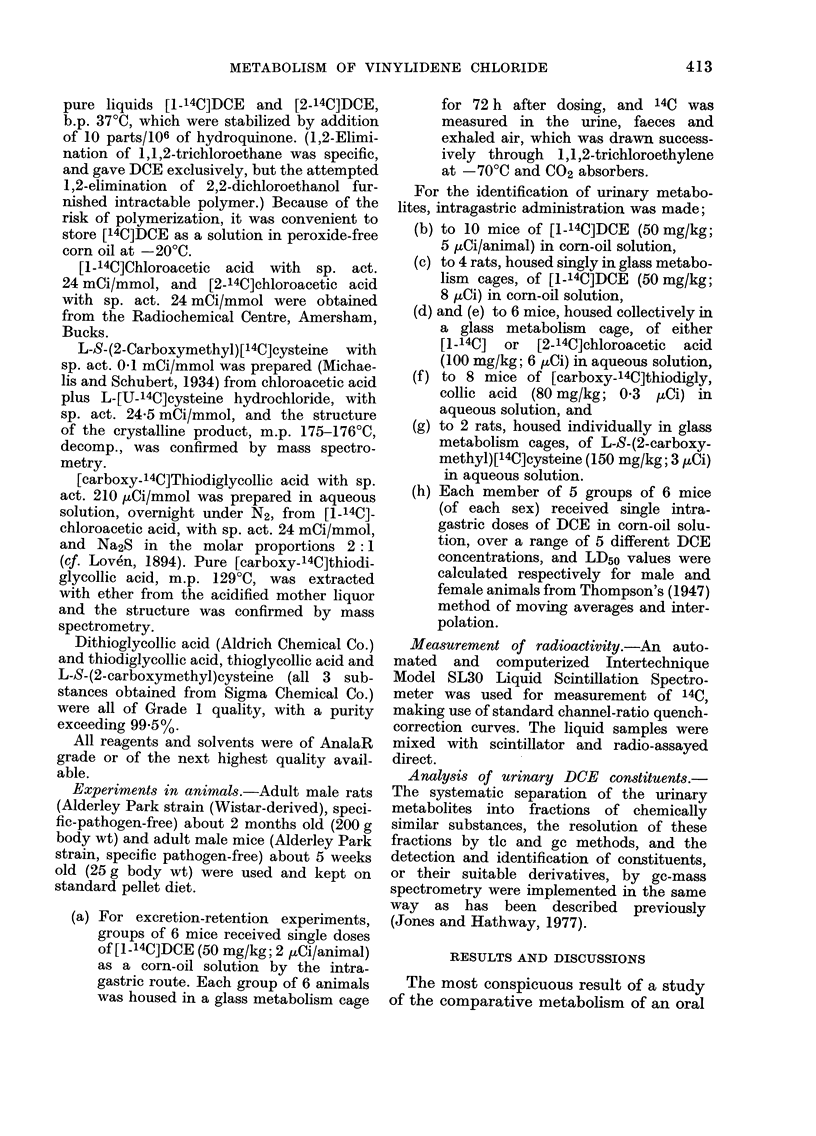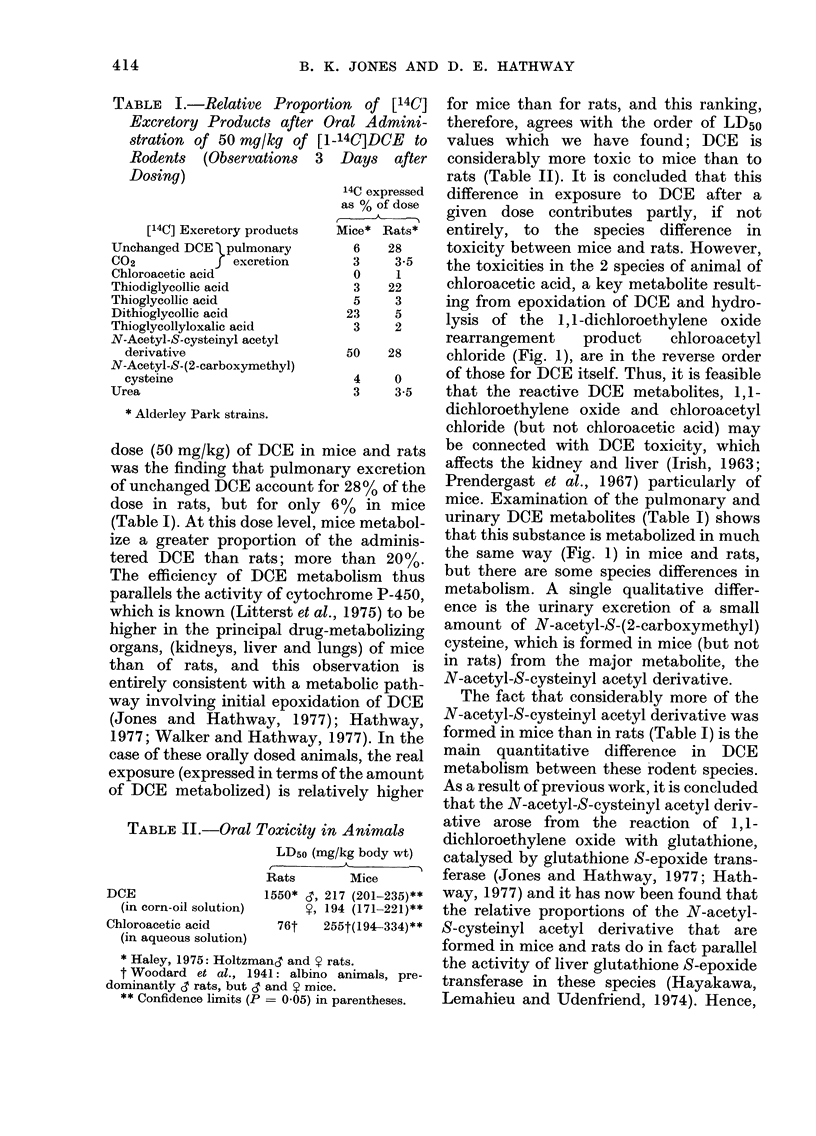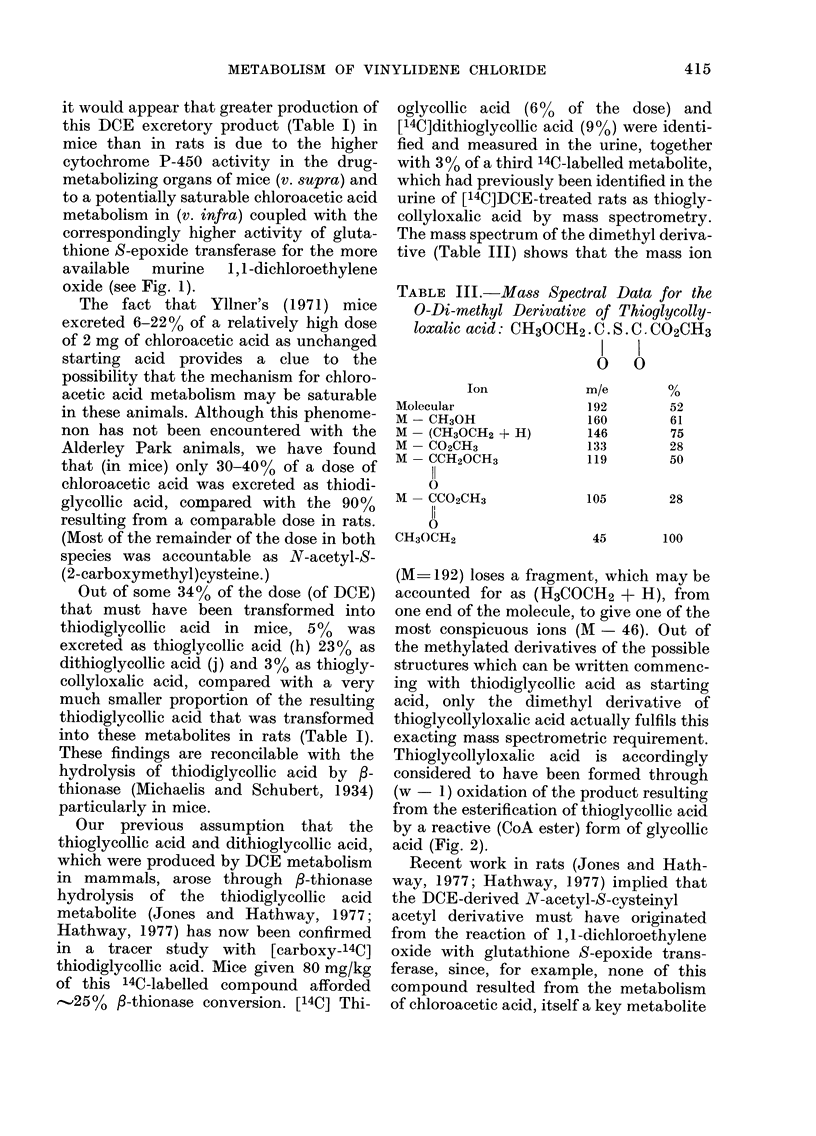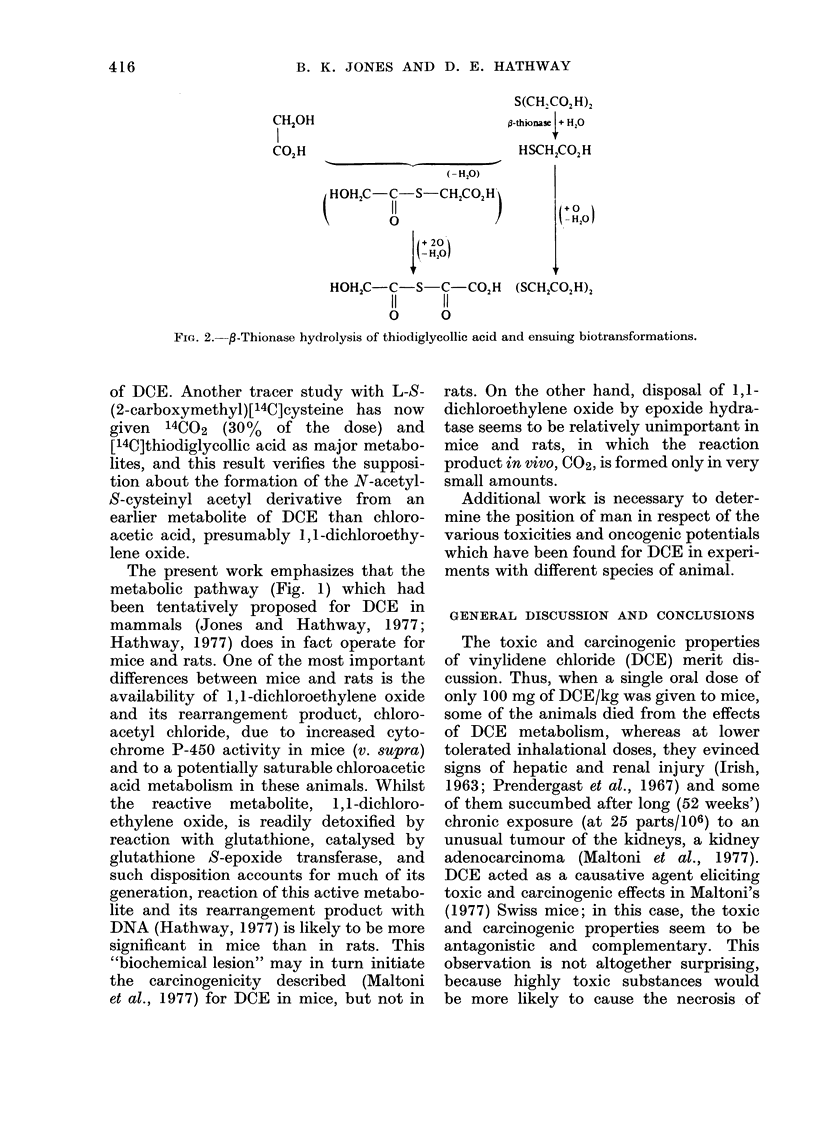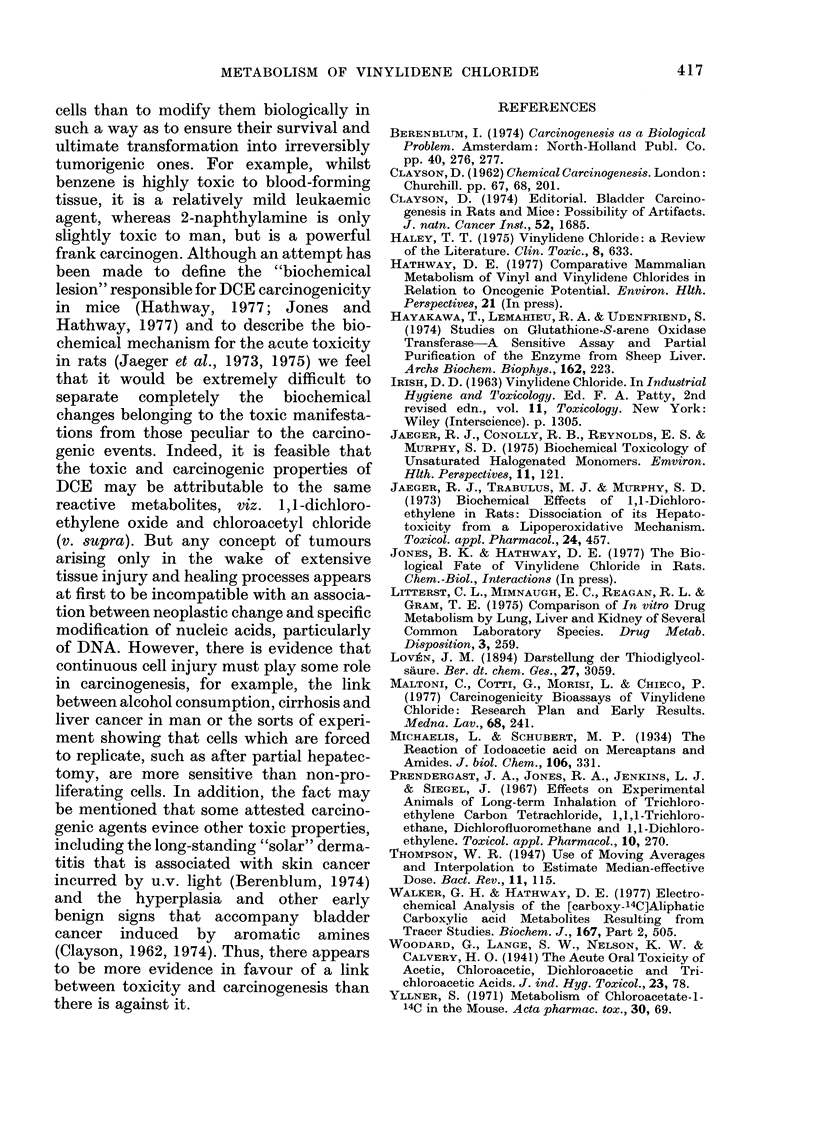# Differences in Metabolism of Vinylidene Chloride Between Mice and Rats

**DOI:** 10.1038/bjc.1978.61

**Published:** 1978-03

**Authors:** B. K. Jones, D. E. Hathway

## Abstract

The present finding that mice metabolize a greater proportion of an oral dose (50 mg/kg) of vinylidence chloride. (1,1 - dichloroethylene, DCE) than rats implies (a) that the efficiency of DCE metabolism follows the known activity of cytochrome P-450 in the organs of these animals, and (b) that, in accordance with the LD_50_ values, the real exposure (expressed as the amount of DCE metabolized) is relatively higher for orally dosed mice than rats, and (c) that DCE carcinogenicity would appear to be more likely in mice than rats.

Mice metabolize DCE simiarly to rats (Jones and Hathway, 1977) but there are some differences. Thus, qualitatively, treated mice (but not rats) excrete a small amount of *N*-acetyl-*S*-(2carboxymethyl)cysteine. Quantitatively, (i) the relative proportions of the *N*-acetyl-*S*-(2-cysteinyl acetyl derivative that are formed in mice and rats parallel the activity of liver glutathione-*S*-epoxide transferase in these rodents, and (ii) there are marked differences in the proportions of DCE metabolites belonging to the chloroacetic acid branch of the metabolic pathway. Furthermore, the previously assumed β-thionase hydrolysis of thiodiglycollic acid (Jones and Hathway, 1977) is now established *in vivo*, and the possible biogenesis of the *N*-acetyl-*S*-cysteinyl acetyl derivative is verified by another tracer study. The conclusion is drawn that the DCE metabolites, 1,1-dichloroethylene oxide and chloroacetyl chloride, may be important to murine DCE carcinogenicity.


*Br. J. Cancer* (1978) **37**, 411

### DIFFERENCES IN METABOLISM OF VINYLIDENE CHLORIDE BETWEEN MICE AND RATS

B. K. JONES AND D. E. HATHWAY

*From the Imperial Chemical Industries, Central Toxicology Laboratory, Alderley Park, Cheshire SK10 4TJ*

Received 18 October 1977 Accepted 25 November 1977

**Summary.—The present finding that mice metabolize a greater proportion of an oral dose (50 mg/kg) of vinylidene chloride. (1,1-dichloroethylene, DCE) than rats implies (a) that the efficiency of DCE metabolism follows the known activity of cytochrome P-450 in the organs of these animals, and (b) that, in accordance with the $LD_{50}$ values, the real exposure (expressed as the amount of DCE metabolized) is relatively higher for orally dosed mice than rats, and (c) that DCE carcinogenicity would appear to be more likely in mice than rats.**

**Mice metabolize DCE similarly to rats (Jones and Hathway, 1977) but there are some differences. Thus, qualitatively, treated mice (but not rats) excrete a small amount of *N*-acetyl-*S*-(2-carboxymethyl)cysteine. Quantitatively, (i) the relative proportions of the *N*-acetyl-*S*-cysteinyl acetyl derivative that are formed in mice and rats parallel the activity of liver glutathione-*S*-epoxide transferase in these rodents, and (ii) there are marked differences in the proportions of DCE metabolites belonging to the chloroacetic acid branch of the metabolic pathway. Furthermore, the previously assumed β-thionase hydrolysis of thiodiglycollic acid (Jones and Hathway, 1977) is now established *in vivo*, and the possible biogenesis of the *N*-acetyl-*S*-cysteinyl acetyl derivative is verified by another tracer study. The conclusion is drawn that the DCE metabolites, 1,1-dichloroethylene oxide and chloroacetyl chloride, may be important to murine DCE carcinogenicity.**

WIDESPREAD use of the polymer of vinylidene chloride* (1,1-dichloroethylene, DCE) for packaging film and for coating other packaging materials, and the recent discovery of DCE tumorigenicity in the kidneys of mice (Maltoni *et al.*, 1977) but not in rats, warrants a systematic search for possible species differences in DCE metabolism which might account for the species susceptibility observed.

Previous work (Jones and Hathway, 1977; Walker and Hathway, 1977) on the metabolism of DCE (Fig. 1(a)) in rats showed that:

(i) thiodiglycollic acid (g) and an *N*-acetyl-*S*-cysteinyl acetyl derivative (e) (where R is considered to be OH and R′ is unknown) where the major urinary metabolites associated with substantial amounts of chloroacetic acid (b) dithioglycollic acid (j) and thioglycollic acid (h),

(ii) chloroacetic acid (b) a key metabolite of DCE (a) biotransformation, afforded *in vivo* several metabolites in common with DCE, and

(iii) transformation of DCE (a) into chloroacetic acid (b) involved migration of one Cl atom and the loss of the other one.

The experimental evidence implied that the *N*-acetyl-*S*-cysteinyl acetyl derivative (e) arises through the reaction of 1,1-dichloroethylene oxide with glutathione, a reaction catalysed by glutathione *S*-epoxide transferase.

The present paper describes the results of an investigation of DCE metabolism in mice *vis-à-vis* the previous one in rats

* Known commercially as VDC.

FIG. 1.—Metabolic pathway for vinylidene chloride in mammals (see Introductory Section, for the key to this scheme).

(Jones and Hathway, 1977) and attempts to relate differences to the susceptibility of the mouse to DCE.

### MATERIALS AND METHODS

*Chemicals.*—Vinylidene chloride was supplied by Imperial Chemical Industries Limited, Mond Division, Runcorn, Cheshire. Before each experiment, stabilizer was removed by washing with alkali and water.

[1-$^{14}$C]DCE and [2-$^{14}$C)DCE with sp. act. 1·0 mCi/mmol, and with a chemical and radiochemical purity >99·0%, were synthesized from $^{14}CO_2$ by our colleagues Mr D. C. Greenslade and Dr J. A. Heslop of Imperial Chemical Industries Limited, Petrochemicals Division, Billingham, Cleveland. Whereas in the case of [1-$^{14}$C]DCE, $^{14}CO_2$ was converted into [1-$^{14}$C]acetic acid by the Grignard reaction with methyl iodide, in that of [2-$^{14}$C]DCE, $^{14}CO_2$ was reduced to [$^{14}$C]-methanol, which was transformed by a reaction sequence involving [$^{14}$C]methyl iodide and [2-$^{14}$C]acetonitrile into [2-$^{14}$C]-acetic acid. [1-$^{14}$C] and [2-$^{14}$C]acetic acids respectively were then transformed through the variously labelled forms of the reaction sequence, dichloroacetic acid, 2,2-dichloroethanol and 1,1,2-trichloroethane, into the pure liquids [1-$^{14}$C]DCE and [2-$^{14}$C]DCE, b.p. 37°C, which were stabilized by addition of 10 parts/$10^6$ of hydroquinone. (1,2-Elimination of 1,1,2-trichloroethane was specific, and gave DCE exclusively, but the attempted 1,2-elimination of 2,2-dichloroethanol furnished intractable polymer.) Because of the risk of polymerization, it was convenient to store [$^{14}$C]DCE as a solution in peroxide-free corn oil at −20°C.

[1-$^{14}$C]Chloroacetic acid with sp. act. 24 mCi/mmol, and [2-$^{14}$C]chloroacetic acid with sp. act. 24 mCi/mmol were obtained from the Radiochemical Centre, Amersham, Bucks.

L-*S*-(2-Carboxymethyl)[$^{14}$C]cysteine with sp. act. 0·1 mCi/mmol was prepared (Michaelis and Schubert, 1934) from chloroacetic acid plus L-[U-$^{14}$C]cysteine hydrochloride, with sp. act. 24·5 mCi/mmol, and the structure of the crystalline product, m.p. 175–176°C, decomp., was confirmed by mass spectrometry.

[carboxy-$^{14}$C]Thiodiglycollic acid with sp. act. 210 $\mu$Ci/mmol was prepared in aqueous solution, overnight under $N_2$, from [1-$^{14}$C]-chloroacetic acid, with sp. act. 24 mCi/mmol, and $Na_2S$ in the molar proportions 2 : 1 (*cf.* Lovén, 1894). Pure [carboxy-$^{14}$C]thiodiglycollic acid, m.p. 129°C, was extracted with ether from the acidified mother liquor and the structure was confirmed by mass spectrometry.

Dithioglycollic acid (Aldrich Chemical Co.) and thiodiglycollic acid, thioglycollic acid and L-*S*-(2-carboxymethyl)cysteine (all 3 substances obtained from Sigma Chemical Co.) were all of Grade 1 quality, with a purity exceeding 99·5%.

All reagents and solvents were of AnalaR grade or of the next highest quality available.

*Experiments in animals.*—Adult male rats (Alderley Park strain (Wistar-derived), specific-pathogen-free) about 2 months old (200 g body wt) and adult male mice (Alderley Park strain, specific pathogen-free) about 5 weeks old (25 g body wt) were used and kept on standard pellet diet.

(a) For excretion-retention experiments, groups of 6 mice received single doses of [1-$^{14}$C]DCE (50 mg/kg; 2 $\mu$Ci/animal) as a corn-oil solution by the intragastric route. Each group of 6 animals was housed in a glass metabolism cage for 72 h after dosing, and $^{14}$C was measured in the urine, faeces and exhaled air, which was drawn successively through 1,1,2-trichloroethylene at −70°C and $CO_2$ absorbers.

For the identification of urinary metabolites, intragastric administration was made;

(b) to 10 mice of [1-$^{14}$C]DCE (50 mg/kg; 5 $\mu$Ci/animal) in corn-oil solution,
(c) to 4 rats, housed singly in glass metabolism cages, of [1-$^{14}$C]DCE (50 mg/kg; 8 $\mu$Ci) in corn-oil solution,
(d) and (e) to 6 mice, housed collectively in a glass metabolism cage, of either [1-$^{14}$C] or [2-$^{14}$C]chloroacetic acid (100 mg/kg; 6 $\mu$Ci) in aqueous solution,
(f) to 8 mice of [carboxy-$^{14}$C]thiodigly, collic acid (80 mg/kg; 0·3 $\mu$Ci) in aqueous solution, and
(g) to 2 rats, housed individually in glass metabolism cages, of L-*S*-(2-carboxymethyl)[$^{14}$C]cysteine (150 mg/kg; 3 $\mu$Ci) in aqueous solution.
(h) Each member of 5 groups of 6 mice (of each sex) received single intragastric doses of DCE in corn-oil solution, over a range of 5 different DCE concentrations, and $LD_{50}$ values were calculated respectively for male and female animals from Thompson's (1947) method of moving averages and interpolation.

*Measurement of radioactivity.*—An automated and computerized Intertechnique Model SL30 Liquid Scintillation Spectrometer was used for measurement of $^{14}$C, making use of standard channel-ratio quench-correction curves. The liquid samples were mixed with scintillator and radio-assayed direct.

*Analysis of urinary DCE constituents.*—The systematic separation of the urinary metabolites into fractions of chemically similar substances, the resolution of these fractions by tlc and gc methods, and the detection and identification of constituents, or their suitable derivatives, by gc-mass spectrometry were implemented in the same way as has been described previously (Jones and Hathway, 1977).

### RESULTS AND DISCUSSIONS

The most conspicuous result of a study of the comparative metabolism of an oral

TABLE I.—*Relative Proportion of* [$^{14}C$] *Excretory Products after Oral Administration of* 50 *mg/kg of* [1-$^{14}C$]*DCE to Rodents* (*Observations* 3 *Days after Dosing*)

| [$^{14}C$] Excretory products | $^{14}C$ expressed as % of dose: Mice* | Rats* |
|---|---|---|
| Unchanged DCE } pulmonary excretion | 6 | 28 |
| $CO_2$ } pulmonary excretion | 3 | 3·5 |
| Chloroacetic acid | 0 | 1 |
| Thiodiglycollic acid | 3 | 22 |
| Thioglycollic acid | 5 | 3 |
| Dithioglycollic acid | 23 | 5 |
| Thioglycollyloxalic acid | 3 | 2 |
| N-Acetyl-S-cysteinyl acetyl derivative | 50 | 28 |
| N-Acetyl-S-(2-carboxymethyl) cysteine | 4 | 0 |
| Urea | 3 | 3·5 |

* Alderley Park strains.

dose (50 mg/kg) of DCE in mice and rats was the finding that pulmonary excretion of unchanged DCE account for 28% of the dose in rats, but for only 6% in mice (Table I). At this dose level, mice metabolize a greater proportion of the administered DCE than rats; more than 20%. The efficiency of DCE metabolism thus parallels the activity of cytochrome P-450, which is known (Litterst *et al.*, 1975) to be higher in the principal drug-metabolizing organs, (kidneys, liver and lungs) of mice than of rats, and this observation is entirely consistent with a metabolic pathway involving initial epoxidation of DCE (Jones and Hathway, 1977); Hathway, 1977; Walker and Hathway, 1977). In the case of these orally dosed animals, the real exposure (expressed in terms of the amount of DCE metabolized) is relatively higher for mice than for rats, and this ranking, therefore, agrees with the order of $LD_{50}$ values which we have found; DCE is considerably more toxic to mice than to rats (Table II). It is concluded that this difference in exposure to DCE after a given dose contributes partly, if not entirely, to the species difference in toxicity between mice and rats. However, the toxicities in the 2 species of animal of chloroacetic acid, a key metabolite resulting from epoxidation of DCE and hydrolysis of the 1,1-dichloroethylene oxide rearrangement product chloroacetyl chloride (Fig. 1), are in the reverse order of those for DCE itself. Thus, it is feasible that the reactive DCE metabolites, 1,1-dichloroethylene oxide and chloroacetyl chloride (but not chloroacetic acid) may be connected with DCE toxicity, which affects the kidney and liver (Irish, 1963; Prendergast *et al.*, 1967) particularly of mice. Examination of the pulmonary and urinary DCE metabolites (Table I) shows that this substance is metabolized in much the same way (Fig. 1) in mice and rats, but there are some species differences in metabolism. A single qualitative difference is the urinary excretion of a small amount of *N*-acetyl-*S*-(2-carboxymethyl) cysteine, which is formed in mice (but not in rats) from the major metabolite, the *N*-acetyl-*S*-cysteinyl acetyl derivative.

TABLE II.—*Oral Toxicity in Animals*

| | $LD_{50}$ (mg/kg body wt): Rats | Mice |
|---|---|---|
| DCE (in corn-oil solution) | 1550* | ♂, 217 (201–235)**<br>♀, 194 (171–221)** |
| Chloroacetic acid (in aqueous solution) | 76† | 255†(194–334)** |

* Haley, 1975: Holtzman♂ and ♀ rats.

† Woodard *et al.*, 1941: albino animals, predominantly ♂ rats, but ♂ and ♀ mice.

** Confidence limits ($P = 0·05$) in parentheses.

The fact that considerably more of the *N*-acetyl-*S*-cysteinyl acetyl derivative was formed in mice than in rats (Table I) is the main quantitative difference in DCE metabolism between these rodent species. As a result of previous work, it is concluded that the *N*-acetyl-*S*-cysteinyl acetyl derivative arose from the reaction of 1,1-dichloroethylene oxide with glutathione, catalysed by glutathione *S*-epoxide transferase (Jones and Hathway, 1977; Hathway, 1977) and it has now been found that the relative proportions of the *N*-acetyl-*S*-cysteinyl acetyl derivative that are formed in mice and rats do in fact parallel the activity of liver glutathione *S*-epoxide transferase in these species (Hayakawa, Lemahieu and Udenfriend, 1974). Hence, it would appear that greater production of this DCE excretory product (Table I) in mice than in rats is due to the higher cytochrome P-450 activity in the drug-metabolizing organs of mice (*v. supra*) and to a potentially saturable chloroacetic acid metabolism in (*v. infra*) coupled with the correspondingly higher activity of glutathione *S*-epoxide transferase for the more available murine 1,1-dichloroethylene oxide (see Fig. 1).

The fact that Yllner's (1971) mice excreted 6–22% of a relatively high dose of 2 mg of chloroacetic acid as unchanged starting acid provides a clue to the possibility that the mechanism for chloroacetic acid metabolism may be saturable in these animals. Although this phenomenon has not been encountered with the Alderley Park animals, we have found that (in mice) only 30–40% of a dose of chloroacetic acid was excreted as thiodiglycollic acid, compared with the 90% resulting from a comparable dose in rats. (Most of the remainder of the dose in both species was accountable as *N*-acetyl-*S*-(2-carboxymethyl)cysteine.)

Out of some 34% of the dose (of DCE) that must have been transformed into thiodiglycollic acid in mice, 5% was excreted as thioglycollic acid (h) 23% as dithioglycollic acid (j) and 3% as thioglycollyloxalic acid, compared with a very much smaller proportion of the resulting thiodiglycollic acid that was transformed into these metabolites in rats (Table I). These findings are reconcilable with the hydrolysis of thiodiglycollic acid by $\beta$-thionase (Michaelis and Schubert, 1934) particularly in mice.

Our previous assumption that the thioglycollic acid and dithioglycollic acid, which were produced by DCE metabolism in mammals, arose through $\beta$-thionase hydrolysis of the thiodiglycollic acid metabolite (Jones and Hathway, 1977; Hathway, 1977) has now been confirmed in a tracer study with [carboxy-$^{14}$C] thiodiglycollic acid. Mice given 80 mg/kg of this $^{14}$C-labelled compound afforded ~25% $\beta$-thionase conversion. [$^{14}$C] Thioglycollic acid (6% of the dose) and [$^{14}$C]dithioglycollic acid (9%) were identified and measured in the urine, together with 3% of a third $^{14}$C-labelled metabolite, which had previously been identified in the urine of [$^{14}$C]DCE-treated rats as thioglycollyloxalic acid by mass spectrometry. The mass spectrum of the dimethyl derivative (Table III) shows that the mass ion

TABLE III.—*Mass Spectral Data for the O-Di-methyl Derivative of Thioglycollyloxalic acid:* $CH_3OCH_2.\underset{\underset{O}{|}}{C}.S.\underset{\underset{O}{|}}{C}.CO_2CH_3$

| Ion | m/e | % |
|---|---|---|
| Molecular | 192 | 52 |
| M − $CH_3OH$ | 160 | 61 |
| M − ($CH_3OCH_2$ + H) | 146 | 75 |
| M − $CO_2CH_3$ | 133 | 28 |
| M − $\underset{\underset{O}{\|}}{C}CH_2OCH_3$ | 119 | 50 |
| M − $\underset{\underset{O}{\|}}{C}CO_2CH_3$ | 105 | 28 |
| $CH_3OCH_2$ | 45 | 100 |

(M=192) loses a fragment, which may be accounted for as ($H_3COCH_2$ + H), from one end of the molecule, to give one of the most conspicuous ions (M − 46). Out of the methylated derivatives of the possible structures which can be written commencing with thiodiglycollic acid as starting acid, only the dimethyl derivative of thioglycollyloxalic acid actually fulfils this exacting mass spectrometric requirement. Thioglycollyloxalic acid is accordingly considered to have been formed through (w − 1) oxidation of the product resulting from the esterification of thioglycollic acid by a reactive (CoA ester) form of glycollic acid (Fig. 2).

Recent work in rats (Jones and Hathway, 1977; Hathway, 1977) implied that the DCE-derived *N*-acetyl-*S*-cysteinyl acetyl derivative must have originated from the reaction of 1,1-dichloroethylene oxide with glutathione *S*-epoxide transferase, since, for example, none of this compound resulted from the metabolism of chloroacetic acid, itself a key metabolite

FIG. 2.—β-Thionase hydrolysis of thiodiglycollic acid and ensuing biotransformations.

of DCE. Another tracer study with L-*S*-(2-carboxymethyl)[$^{14}$C]cysteine has now given $^{14}CO_2$ (30% of the dose) and [$^{14}$C]thiodiglycollic acid as major metabolites, and this result verifies the supposition about the formation of the *N*-acetyl-*S*-cysteinyl acetyl derivative from an earlier metabolite of DCE than chloroacetic acid, presumably 1,1-dichloroethylene oxide.

The present work emphasizes that the metabolic pathway (Fig. 1) which had been tentatively proposed for DCE in mammals (Jones and Hathway, 1977; Hathway, 1977) does in fact operate for mice and rats. One of the most important differences between mice and rats is the availability of 1,1-dichloroethylene oxide and its rearrangement product, chloroacetyl chloride, due to increased cytochrome P-450 activity in mice (*v. supra*) and to a potentially saturable chloroacetic acid metabolism in these animals. Whilst the reactive metabolite, 1,1-dichloroethylene oxide, is readily detoxified by reaction with glutathione, catalysed by glutathione *S*-epoxide transferase, and such disposition accounts for much of its generation, reaction of this active metabolite and its rearrangement product with DNA (Hathway, 1977) is likely to be more significant in mice than in rats. This "biochemical lesion" may in turn initiate the carcinogenicity described (Maltoni *et al.*, 1977) for DCE in mice, but not in rats. On the other hand, disposal of 1,1-dichloroethylene oxide by epoxide hydratase seems to be relatively unimportant in mice and rats, in which the reaction product *in vivo*, $CO_2$, is formed only in very small amounts.

Additional work is necessary to determine the position of man in respect of the various toxicities and oncogenic potentials which have been found for DCE in experiments with different species of animal.

### GENERAL DISCUSSION AND CONCLUSIONS

The toxic and carcinogenic properties of vinylidene chloride (DCE) merit discussion. Thus, when a single oral dose of only 100 mg of DCE/kg was given to mice, some of the animals died from the effects of DCE metabolism, whereas at lower tolerated inhalational doses, they evinced signs of hepatic and renal injury (Irish, 1963; Prendergast *et al.*, 1967) and some of them succumbed after long (52 weeks') chronic exposure (at 25 parts/$10^6$) to an unusual tumour of the kidneys, a kidney adenocarcinoma (Maltoni *et al.*, 1977). DCE acted as a causative agent eliciting toxic and carcinogenic effects in Maltoni's (1977) Swiss mice; in this case, the toxic and carcinogenic properties seem to be antagonistic and complementary. This observation is not altogether surprising, because highly toxic substances would be more likely to cause the necrosis of cells than to modify them biologically in such a way as to ensure their survival and ultimate transformation into irreversibly tumorigenic ones. For example, whilst benzene is highly toxic to blood-forming tissue, it is a relatively mild leukaemic agent, whereas 2-naphthylamine is only slightly toxic to man, but is a powerful frank carcinogen. Although an attempt has been made to define the "biochemical lesion" responsible for DCE carcinogenicity in mice (Hathway, 1977; Jones and Hathway, 1977) and to describe the biochemical mechanism for the acute toxicity in rats (Jaeger *et al.*, 1973, 1975) we feel that it would be extremely difficult to separate completely the biochemical changes belonging to the toxic manifestations from those peculiar to the carcinogenic events. Indeed, it is feasible that the toxic and carcinogenic properties of DCE may be attributable to the same reactive metabolites, *viz.* 1,1-dichloroethylene oxide and chloroacetyl chloride (*v. supra*). But any concept of tumours arising only in the wake of extensive tissue injury and healing processes appears at first to be incompatible with an association between neoplastic change and specific modification of nucleic acids, particularly of DNA. However, there is evidence that continuous cell injury must play some role in carcinogenesis, for example, the link between alcohol consumption, cirrhosis and liver cancer in man or the sorts of experiment showing that cells which are forced to replicate, such as after partial hepatectomy, are more sensitive than non-proliferating cells. In addition, the fact may be mentioned that some attested carcinogenic agents evince other toxic properties, including the long-standing "solar" dermatitis that is associated with skin cancer incurred by u.v. light (Berenblum, 1974) and the hyperplasia and other early benign signs that accompany bladder cancer induced by aromatic amines (Clayson, 1962, 1974). Thus, there appears to be more evidence in favour of a link between toxicity and carcinogenesis than there is against it.

### REFERENCES

BERENBLUM, I. (1974) *Carcinogenesis as a Biological Problem.* Amsterdam: North-Holland Publ. Co. pp. 40, 276, 277.

CLAYSON, D. (1962) *Chemical Carcinogenesis.* London: Churchill. pp. 67, 68, 201.

CLAYSON, D. (1974) Editorial. Bladder Carcinogenesis in Rats and Mice: Possibility of Artifacts. *J. natn. Cancer Inst.*, **52**, 1685.

HALEY, T. T. (1975) Vinylidene Chloride: a Review of the Literature. *Clin. Toxic.*, **8**, 633.

HATHWAY, D. E. (1977) Comparative Mammalian Metabolism of Vinyl and Vinylidene Chlorides in Relation to Oncogenic Potential. *Environ. Hlth. Perspectives*, **21** (In press).

HAYAKAWA, T., LEMAHIEU, R. A. & UDENFRIEND, S. (1974) Studies on Glutathione-*S*-arene Oxidase Transferase—A Sensitive Assay and Partial Purification of the Enzyme from Sheep Liver. *Archs Biochem. Biophys.*, **162**, 223.

IRISH, D. D. (1963) Vinylidene Chloride. In *Industrial Hygiene and Toxicology.* Ed. F. A. Patty, 2nd revised edn., vol. **11**, *Toxicology.* New York: Wiley (Interscience). p. 1305.

JAEGER, R. J., CONOLLY, R. B., REYNOLDS, E. S. & MURPHY, S. D. (1975) Biochemical Toxicology of Unsaturated Halogenated Monomers. *Emviron. Hlth. Perspectives*, **11**, 121.

JAEGER, R. J., TRABULUS, M. J. & MURPHY, S. D. (1973) Biochemical Effects of 1,1-Dichloroethylene in Rats: Dissociation of its Hepatotoxicity from a Lipoperoxidative Mechanism. *Toxicol. appl. Pharmacol.*, **24**, 457.

JONES, B. K. & HATHWAY, D. E. (1977) The Biological Fate of Vinylidene Chloride in Rats. *Chem.-Biol., Interactions* (In press).

LITTERST, C. L., MIMNAUGH, E. C., REAGAN, R. L. & GRAM, T. E. (1975) Comparison of *In vitro* Drug Metabolism by Lung, Liver and Kidney of Several Common Laboratory Species. *Drug Metab. Disposition*, **3**, 259.

LOVÉN, J. M. (1894) Darstellung der Thiodiglycolsäure. *Ber. dt. chem. Ges.*, **27**, 3059.

MALTONI, C., COTTI, G., MORISI, L. & CHIECO, P. (1977) Carcinogenicity Bioassays of Vinylidene Chloride: Research Plan and Early Results. *Medna. Lav.*, **68**, 241.

MICHAELIS, L. & SCHUBERT, M. P. (1934) The Reaction of Iodoacetic acid on Mercaptans and Amides. *J. biol. Chem.*, **106**, 331.

PRENDERGAST, J. A., JONES, R. A., JENKINS, L. J. & SIEGEL, J. (1967) Effects on Experimental Animals of Long-term Inhalation of Trichloroethylene Carbon Tetrachloride, 1,1,1-Trichloroethane, Dichlorofluoromethane and 1,1-Dichloroethylene. *Toxicol. appl. Pharmacol.*, **10**, 270.

THOMPSON, W. R. (1947) Use of Moving Averages and Interpolation to Estimate Median-effective Dose. *Bact. Rev.*, **11**, 115.

WALKER, G. H. & HATHWAY, D. E. (1977) Electrochemical Analysis of the [carboxy-$^{14}$C]Aliphatic Carboxylic acid Metabolites Resulting from Tracer Studies. *Biochem. J.*, **167**, Part 2, 505.

WOODARD, G., LANGE, S. W., NELSON, K. W. & CALVERY, H. O. (1941) The Acute Oral Toxicity of Acetic, Chloroacetic, Dichloroacetic and Trichloroacetic Acids. *J. ind. Hyg. Toxicol.*, **23**, 78.

YLLNER, S. (1971) Metabolism of Chloroacetate-1-$^{14}$C in the Mouse. *Acta pharmac. tox.*, **30**, 69.